# Expression of AEG-1 mRNA and protein in colorectal cancer patients and colon cancer cell lines

**DOI:** 10.1186/1479-5876-10-109

**Published:** 2012-05-29

**Authors:** Sebastian Gnosa, Yang-Mei Shen, Chao-Jie Wang, Hong Zhang, Johannes Stratmann, Gunnar Arbman, Xiao-Feng Sun

**Affiliations:** 1 Division of Oncology, Department of Clinical and Experimental Medicine, Faculty of Health Sciences, University of Linköping, Linköping, Sweden; 2 Department of Pathology, West China Second University Hospital, Sichuan University, Chengdu, People's Republic of China; 3 Division of Tumour Biology, Systems Biology Research Centre, School of Life Sciences, Skövde University, Skövde, Sweden; 4 Department of Surgery, Vrinnevi Hospital, Norrköping, Sweden; 5 Division of Oncology, Department of Clinical and Experimental Medicine, Faculty of Health Sciences, Country Council of Östergötland, University of Linköping, S-581 85, Linköping, Sweden

## Abstract

**Background:**

Astrocyte elevated gene 1 (AEG-1), an important oncogene, has been shown to be overexpressed in several types of cancers. In colorectal cancer (CRC), the protein level of AEG-1 is up-regulated in tumour tissue compared to normal mucosa, showing prognostic significance. Since little is known about the transcriptional level of AEG-1 expression and its biological pathway in CRC the aim of the present study was to examine the relationship of AEG-1 mRNA expression, the protein level and clinicopathological variables as well as its biology pathway in CRC.

**Material and methods:**

The mRNA expression of AEG-1 was analysed by qPCR in fresh frozen patient samples including 156 primary tumours, along with the corresponding normal mucosa, and in five colon cancer cell lines, SW480, SW620, KM12C, KM12SM and KM12L4a. AEG-1 protein expression was investigated by immunohistochemistry in paraffin-embedded materials from 74 distant normal mucosa, 107 adjacent mucosa, 158 primary tumour, 35 lymph node metastasis and 9 liver metastasis samples. In addition, the AEG-1 protein expression was elucidated in the cell lines by Western blot.

**Results:**

The lymph node metastatic cell line SW620 had a significantly higher AEG-1 mRNA (0.27 ± 0.02) expression compared to the primary tumour cell line SW480 (0.17 ± 0.04, *p* = 0.026). AEG-1 expression at the mRNA level and/or the protein level was significantly up-regulated gradually from normal mucosa to primary CRC, and then to lymph node metastasis and finally to liver metastasis (*p* < 0.05). There were significant associations of AEG-1 mRNA expression with tumour location (*p* = 0.047), as well as mRNA and protein expression with the tumour stage (*p* < 0.03). Furthermore AEG-1 protein expression was positively related to biological variables including NF-κB, p73, Rad50 and apoptosis (*p* < 0.05).

**Conclusion:**

AEG-1 is up-regulated, at the mRNA and the protein level, during CRC development and aggressiveness, and is related to tumour location and stage. It may play its role in CRC through the NF-κB signaling pathway.

## Background

Colorectal cancer (CRC) is the third most common cancer world-wide with about 600.000 estimated deaths making CRC the fourth most common cause of cancer death [1]. Only about 5–10% of CRC cases are hereditary [2], while the rest occur sporadically. Risk factors for CRC are large consumption of alcohol, diet, physical inactivity, obesity and smoking [3]. The tumour genesis is a multistep progression and is characterized by a stepwise accumulation of genetic mutations in key signalling pathways [4]. The manner of aggressiveness depends on the deepness of invasion as well as on the ability to migrate to other organs. The treatment of CRC has been improved during the past two decades, with the median duration survival of CRC patients increasing from 12 months to about 18 to 21 months [5]. To further increase survival time it is of high clinical interest to find powerful biomarkers for prevention and to develop new treatment strategies for this disease.

Astrocyte elevated gene-1 (AEG-1), also known as Metadherin (MTDH) and LYRIC, was originally identified as a human immunodeficiency virus-1 (HIV-1) - inducible gene in human fetal astrocytes [6]. In 2004, Brown *et al.* used phage screening to show a MTDH- mediated metastases of mouse breast cancer cells to the lungs, thereby demonstrating the involvement of MTDH in cancer [7]. Further studies have shown that AEG-1 is markedly overexpressed in oesophageal squamous cell carcinoma [8], gastric cancer [9], CRC [10], hepatocellular carcinoma [11], non-small cell lung cancer [12], neuroblastoma [13], breast cancer [7,14], prostate cancer [15] and renal cancer [16], compared to normal cells and the matched non-neoplastic regions. Immunohistochemical and immunofluorescence studies have demonstrated that AEG-1 is localised at the perinuclear region, nuclear rim, cytoplasm and the endoplasmic reticulum [17]. Lee *et al.* (2006) have shown the first activation pathway of AEG-1 in which the oncogene Ha-ras is overexpressed in human adult astrocytes immortalized by simian virus 40 T/t antigen and hTERT followed by an increase in AEG-1 expression. Furthermore the study has shown that this activation is promoted by the PI3K/Akt pathway, leading to the binding of the transcription factor c-Myc to the promoter region of AEG-1 and transcription [18]. The first discovered target of AEG-1 was the transcription factor nuclear factor-kappa B (NF-κB). AEG-1 facilitates the degradation of the NF-κB inhibitor α (IκBα) and this in turn leads to an increased NF-κB-DNA binding activity. NF-κB, once activated in the cytoplasm, migrates into the nucleus and proceeds to activate the expression of several target genes which are anti-apoptotic and pro-proliferative. Furthermore, in HeLa and human malignant glioma cells, after overexpression of AEG-1, it translocates into the nucleus where it physically interacts with the p65 subunit of NF-κB and modulates its function in the nucleus [19].

In the present study, we evaluated the AEG-1 mRNA and protein expression in CRC patient samples and colon cancer cell lines by qPCR, Western blot and immunohistochemistry to investigate the expression status in CRC development, and the association with clinicopathological and biological variables.

## Materials and methods

### Patient materials

The patient samples for qPCR analyses used in this study included the primary tumour and the corresponding normal mucosa from 156 patients. All samples were flash-frozen and stored at −80 °C. No information was available about stage in 2 patients and about differentiation in 1 patient. Paraffin-embedded material used for immunohistochemistry included 74 distant normal colorectal mucosa samples, which were histologically free from tumour (29 corresponding to the primary tumours, *i.e.* distant normal mucosa and primary samples from the same patients) and taken from the margin of distant resection, 107 adjacent normal mucosa samples (normal mucosa adjacent to the corresponding primary tumour), 158 primary colorectal adenocarcinomas, 35 metastases from the regional lymph nodes (20 corresponding to the primary tumours), and 9 metastases from the liver. Information was lacking about location (1 patient), stage (11 patients), and differentiation (6 patients). All patients were diagnosed at Linköping University Hospital and Vrinnevi Hospital in Norrköping between 1983 and 2003. The patient’s gender, age, tumour location, stage and differentiation were obtained from surgical and pathological records (Table1). The data of phosphorylation of NF-κB/p65 at Ser 536, p73 and Rad50 expression, determined by immunohistochemistry and the apoptosis determined by TUNEL assay were taken from previous studies carried out at our laboratory [20-24]. The results were from the same cohort of the patients as the present study, and varied numbers in the different variables (Table2, Table3) are due to missing data.

**Table 1:** Patient and tumour characteristics

**Characteristics**	**Fresh frozen tissue (%)**	**Paraffin-embedded tissue (%)**
Gender		
Male	103 (66)	88 (56)
Female	53 (34)	70 (44)
Age (years)		
<70	48 (32)	59 (37)
≥70	104 (68)	99 (63)
Location		
Colon	98 (63)	77 (49)
Rectum	57 (37)	81 (51)
Stage		
I	20 (14)	25 (16)
II	73 (50)	61 (39)
III	34 (23)	40 (25)
IV	18 (12)	31 (20)
Differentiation		
Well/moderately	110 (73)	107 (68)
Poorly	40 (27)	50 (32)

Varied number in the different variables is due to missing data.

**Table 2:** AEG-1 cytoplasmic expression in the primary colorectal cancer in relation to biological variables

	**AEG-1 expression in the cytoplasm**		
	**Low (%)**	**High (%)**	***P***
NF-κB p-p65 Ser536			0.002
Low	42 (47)	47 (53)	
High	8 (19)	35 (81)	
p73			0.033
Low	5 (83)	1 (17)	
High	4 (31)	9 (69)	
Rad50			0.024
Low	40 (45)	48 (55)	
High	12 (26)	35 (74)	
Apoptosis			0.006
Low	13 (87)	2 (13)	
High	2 (29)	5 (71)	

Varied number in the different variables is due to missing data.

**Table 3:** AEG-1 nuclear expression in the primary colorectal cancer in relation to biological variables

	**AEG-1 expression in the nucleus**		
	**Low (%)**	**High (%)**	***P***
NF-κB p-p65 Ser536			0.034
Low	15 (56)	12 (44)	
High	35 (33)	70 (67)	
p73			0.037
Low	7 (70)	3 (30)	
High	2 (22)	7 (78)	
Rad50			0.024
Low	8 (30)	19 (70)	
High	13 (12)	95 (88)	
Apoptosis			0.277
Low	8 (80)	2 (20)	
High	7 (58)	5 (42)	

Varied number in the different variables is due to missing data.

### Ethics statement

The study was approved by the Regional Ethical Review Board in Linköping and an informed consent document was signed by participants.

### Cell lines

The SW480 and SW620 cell lines were obtained from American Type Culture Collection. SW480 cell line was established from a primary adenocarcinoma of the colon, and the SW620 from a lymph node metastasis, taken from the same patient one year later. Human colon carcinoma cell lines KM12C, KM12SM and KM12L4a were kindly provided by Prof. I.J. Fidler (M.D. Anderson Cancer centre, Houston, TX). The cell line KM12C derived from a patient with stage II colon cancer. The cell line KM12SM is a spontaneous liver metastasis arisen from the injection of KM12C into the Cecum of nude mice. KM12L4a, an experimental liver metastases, is produced by repeated intraspleen injection and harvesting of the liver metastases in nude mice [25,26]. The cell lines were maintained at 37 °C and 5% CO_2_ in Eagles MEM (Sigma-Aldrich, St. Louis, MO), supplemented with 10% heat inactivated fetal bovine serum albumin (GIBCO, Invitrogen, Paisley, UK), 0.5% L-glutamine (GIBCO), 1% of a penicillin and streptomycin cocktail (GIBCO). For the KM12 cell lines 2% vitamin solution (GIBCO) was added. Cells growing exponentially were harvested when 80% confluence was achieved.

### RNA extraction and cDNA preparation

Total RNA was extracted using the TRIzol reagent (Sigma-Aldrich, St. Louis, MO) and RNeasy extract Kit (QIAGEN, Venlo, NL) according to the manufacturer’s instructions. The concentration, purity and integrity of RNA were measured by NanoDrop (Thermo Scientific, Wilmington, DE) and Bioanalyzer Agilent (Agilent Technologies, Santa Clara, CA). For the RT-PCR, the High Capacity cDNA Reverse Transcription Kit (Applied Biosystems, Foster City, CA) was used. 10 μL total RNA was reverse transcribed using the MultiScribe^TM^ Reverse Transcriptase according to the manufacturer's instructions, without an RNase inhibitor in a final volume of 20 μL. The program was the following: 25 °C 10 min, 37 °C 120 min, 85 °C 5 min and 4 °C 120 min (MJ Research PTC-200 Thermo cycler). The cDNA samples were stored at −80 °C.

### qPCR

The relative expression levels of AEG-1 mRNA in CRC patient material and colon cancer cell lines were determined by standard curve with TaqMan™® Gene Expression Fast Master Mix in Applied Biosystems 7900HT Fast Real-Time PCR System and normalized to GAPDH according to the manufacturer’s instructions. Primers and hydrolysis probes were TaqMan™® Gene Expression assays on demand for *AEG-1* (Hs00292707_m1) and *GAPDH* (4352934E) (Applied Biosystems, Foster City, CA). All samples were performed in triplicates. The PCR amplification program was the following: denature 95 °C 20 sec, amplification and quantification program repeated 40 times 95 °C 1 sec and 60 °C 20 sec. In addition, ddH_2_O and a minus RT product as the negative control were analysed for every plate. For statistical analyses between the AEG-1 mRNA expression and clinicophatological variables, the expression level was divided into two groups by the cutoff point of the mean value.

### Western blot

The AEG-1 protein expression was determined by Western blot assay. Protein was extracted by lysis buffer containing 150 mM NaCl, 2% Triton, 0.1% SDS, 50 mM Tris pH8.0 and 10% Protease inhibitor cocktail (Sigma, St Louis, MO) and stored at −20 °C. The protein concentration was determined by the colorimetric BCA protein assay reagent (Pierce, Woburn, MA). Equal amounts of protein (25 μg) for each sample were loaded into pre-cast 7.5% Mini Protean TGX gels (Bio Rad, Hercules, CA) and separated by electrophoresis for 50 min at 200 V. The separated proteins were transferred to a PVDF transfer membrane (Amersham Bioscience/GE Healthcare, Piscataway, NJ) for 55 min at 100 V. Membranes were blocked with 5% milk in TBS supplemented with 0.1% Tween-20 for 1 h at room temperature and incubated with a primary polyclonal antibody rabbit anti-MTDH (1:1000, Sigma Aldrich, St. Louis, MO) over night at 4 °C. The membranes were washed and subsequently incubated with the secondary HRP conjugated polyclonal goat anti-rabbit antibody (1:2000, DAKO Cytomation, Glostrup, Denmark) for 1 h at room temperature. Protein bands were detected using ECL plus Western Blotting Detection System (Amersham Bioscience/GE Healthcare, Piscataway, NJ). To verify equal protein loadings, blots were stripped and re-probed with a rabbit monoclonal anti-β-actin (1:5000, Cell Signalling Technology, Danvers, MA) and a secondary, HRP- conjugated polyclonal goat anti-rabbit antibody (1:2000, DAKO Cytomation, Glostrup, Denmark). AEG-1 expression of the 75, 65 and 35 kDa isoforms in the different cell lines was calculated in relation to β-actin expression.

### Immunohistochemistry

**Figure 1:**
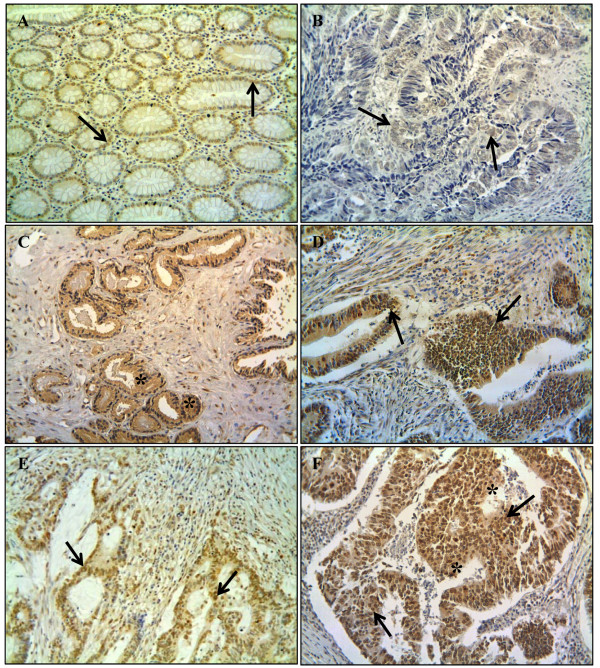
**AEG-1 protein expression determined by immunohistochemistry. A)** Moderate staining in the nucleus of normal mucosa (*arrow* nuclear staining); **B)** weak staining in the nucleus of primary tumour (*arrow* nuclear staining); **C)** strong staining in the cytoplasm of primary tumour (*star symbol* cytoplasmic staining); **D)** strong staining in the nucleus of primary tumour (*arrows* nuclear staining); **E)** moderate staining in the nucleus of lymph node metastasis (*arrow* nuclear staining); and **F)** strong staining in the both nucleus and cytoplasm of liver metastasis (*arrows* nuclear staining, *star symbol* cytoplasmic staining). Original magnification 200x.

Sections from paraffin-embedded tissue blocks were incubated at 60 °C for 12 h, then deparaffinized and hydrated in descending concentrations of ethanol and finally in ddH_2_O. To expose masked epitopes, the sections were microwaved in citrate buffer (pH 6.0) for 2 × 5 min, then kept at room temperature for 30 min, followed by a PBS-wash for 2 min. The activity of endogenous peroxidase was blocked in 3% H_2_O_2_ in methanol and subsequently washed three times in PBS. After blocking with 1.5% blocking serum in PBS for 10 min, the primary rabbit polyclonal anti-MTDH antibody (Sigma Aldrich, St. Louis, MO) was added at 1:150 in antibody diluent (DAKO, Cytomation, Glostrup, Denmark) and then incubated at 4 °C over night. After washing with PBS, a biotinylated secondary anti-rabbit antibody (DAKO Cytomation, Glostrup, Denmark) was added. After 30 min the sections were rinsed with PBS and AB enzyme reagent (ABC Staining System, Santa Cruz Biotechnology), containing avidin, biotinylated horseradish peroxidase and PBS, was added to the slides. The AB enzyme reagent was rinsed off after 30 min with PBS. Peroxidase substrate containing 3,3-diaminobenzidine chromogene, peroxidase substrate buffer and ddH_2_O, was added and incubated for 10 min and subsequently rinsed with water. The sections were then counterstained with haematoxylin. In all runs, negative controls were included, where PBS was used instead of the primary antibody. The degree of immunostaining was reviewed by two independent observers based on the proportion of positively stained cells, the intensity and localisation without knowledge of clinicopathological and biological information. The intensity of staining was classified according to the following criteria: 0 (negative staining), 1 (weak staining), 2 (moderate staining) and 3 (strong staining), and the staining patterns were graded as cytoplasmic or nuclear (Figure1). For statistical analyses, negative and weak stained cases were considered as low expressing group, whereas moderate and strong cases were considered as high expressing group. To avoid artificial effects, cells in areas with necrosis or with poor morphology were not counted.

### Statistical analyses

All data were analysed by the statistics program STATISTICA (SatSoft, Tulsa, OK). Student’s *t*-test was used to test significance between AEG-1 mRNA levels in the different sites of the samples. Student’s *t*-test or one-way ANOVA method was used to analyse the relationship between the AEG-1 mRNA and clinicopathological variables. McNemar’s or Chi-square test was applied to test the significance of the differences in AEG-1 protein expression among normal mucosa, adjacent mucosa, primary tumour, lymph node metastasis and liver metastases as well as the association of AEG-1 expression with clinicopathological/biological variables. Cox’s Proportional Hazard Model was used to test the relationship between the AEG-1 staining and the patient survival. All tests were two sided, and a *p*-value less than 0.05 was considered as significant.

## Results

### AEG-1 mRNA and protein expression in the normal mucosa, primary tumour, lymph node metastasis and liver metastasis of CRC patients

**Figure 2:**
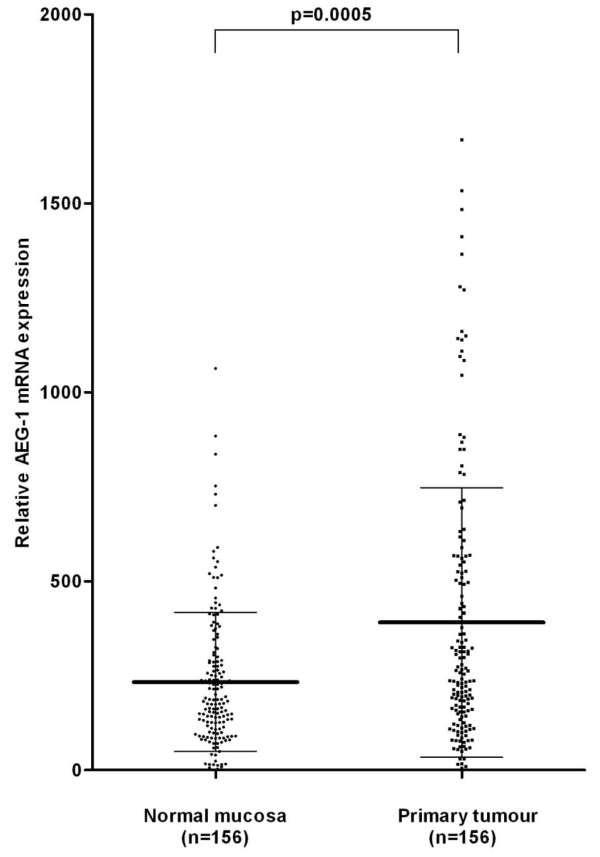
**AEG-1 mRNA expression in normal colorectal mucosa and the corresponding primary colorectal cancer.** The mean value of AEG-1 mRNA expression measured by qPCR increased significantly from normal mucosa (214.98 ± 156.39) to the primary tumour (371.56 ± 348.37, *p* = 0.0005). Bold line and whiskers represent mean ± standard deviation.

AEG-1 mRNA expression was investigated in fresh frozen CRC patient samples including 156 primary tumours and in the corresponding normal mucosa by qPCR. The mean value of AEG-1 mRNA expression was significantly higher in the primary tumour (371.56 ± 348.37) compared to the normal mucosa (214.98 ± 156.39, *p* = 0.0005, Figure2).

**Figure 3:**
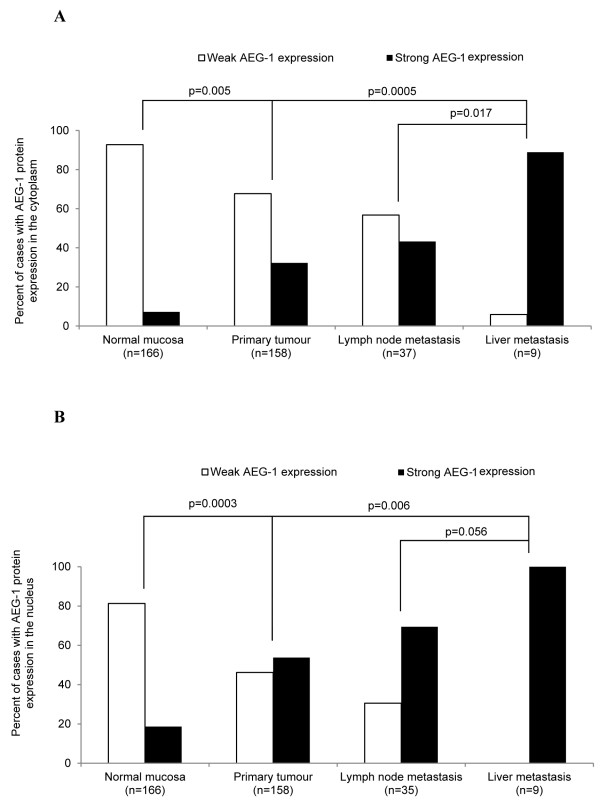
**AEG-1 protein expression in normal colorectal mucosa, primary colorectal cancer and lymph node metastasis.** AEG-1 protein expression was significantly higher in the primary tumour in **(A)** the cytoplasm (*p* = 0.005) and **(B)** the nucleus (*p* = 0.003) compared to the normal mucosa. Furthermore there was a significant higher AEG-1 expression in liver metastases compared to the primary tumours in both **(A)** the cytoplasm (*p* = 0.0005) and **(B)** the nucleus (*p* = 0.006) whereas the expression of AEG-1 was not significantly higher neither in the lymph node metastases in **(A)** the cytoplasm nor **(B)** the nucleus compared to the primary tumours (*p* > 0.05).

Furthermore the AEG-1 protein expression and its localisation were investigated, by immunohistochemistry, in paraffin-embedded materials of the distant normal mucosa, of the adjacent mucosa of primary tumour, of the primary tumour, of lymph node, and of liver metastasis. A positive AEG-1 expression correlated to either cytoplasmic-, or nuclear localisation, or with both (Figure1A-F). As there was no significant difference in AEG-1 staining between the distant and adjacent normal mucosa neither in the cytoplasm (*p* > 0.05) or the nucleus (*p* > 0.05), we combined these two groups as one group called normal mucosa. The frequency of strong AEG-1 staining increased significantly from normal mucosa to primary tumour samples both in the cytoplasm (*p* = 0.005) and in the nucleus (*p* = 0.0003, Figure3A and 3B), in accordance to the findings at the mRNA level. AEG1 expression was slightly increased from primary tumours to lymph node metastases although the increase was not statistically significant (cytoplasm *p* = 0.158, nucleus *p* = 0.081, Figure3A and 3B). AEG-1 expression in the liver metastases was significantly higher compared to the primary tumour and even lymph node metastases, both in the cytoplasm (*p* = 0.0005 and *p* = 0.017, respectively) and in the nucleus (*p* = 0.006 and *p* = 0.056, respectively).

### AEG-1 mRNA and protein expression in colon cancer cell lines

**Figure 4:**
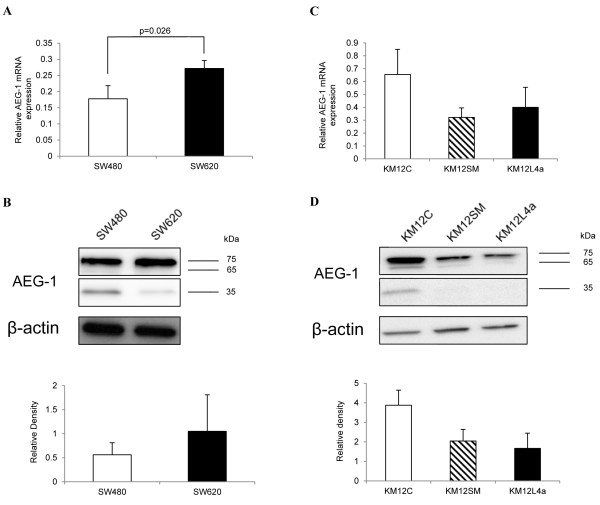
**AEG-1 mRNA and protein expression in the colon cancer cell lines SW480, SW620, KM12C, KM12SM and KM12L4a.** qPCR analyses of AEG-1 expression in the colon cancer cell lines SW480 and SW620 **(A)** and KM12C, KM12SM and KM12L4a **(C)**. The mRNA expression was significantly higher in the metastatic cell line SW620 compared to the parental colon cancer cell line SW480 (*p* = 0.26). AEG-1 protein expression from a representative Western blot image and Densitometric measurement from Western blot images of AEG-1 in the cell lines SW480 and SW620 **(B)** and KM12C, KM12SM and KM12L4a **(D)**. Data are mean ± SD of three independent experiments.

We investigated the AEG-1 mRNA and protein levels from the primary colon cancer cell line SW480 and its lymph node metastatic counterpart SW620 as well as from the primary colon cancer cell line KM12C and their experimental metastatic counterpart cell lines KM12SM and KM12L4a. The metastatic cell line SW620 (0.27 ± 0.02) had a significantly higher AEG-1 mRNA expression compared to the parental primary tumour cell line SW480 (0.17 ± 0.04, *p* = 0.026, Figure4A). Among the three cell lines: KM12C (0.65 ± 0.21), KM12SM (0.32 ± 0.07, *p* > 0.05) and KM12L4a (0.4 ± 0.16, *p* > 0.05), there was no statistical significance in the AEG-1 mRNA expression (Figure4C).

Upon examining the levels of AEG-1 with Western blot, a band of approximately 65 kDa was detected in all of the cell lines (Figure4B and 4D) which is consistent with the molecular weight of AEG-1 [27]. In addition, we detected a protein with a molecular weight of 75 kDa in all of the cell lines, which is consistent with the mono-ubiquitinated from of AEG-1 as well as a band around 35 kDa in the SW480 and KM12C cell line, consistent with an alternatively spliced AEG-1 variant as found before [15,27]. The AEG-1 protein expression showed a similar pattern at the mRNA level in all of the cell lines. A non-statistically significant higher expression was found in the metastatic cell line SW620 compared to the parental primary colon cancer cell line SW480 (*p* > 0.05, Figure4B) and a non-significantly higher expression in the primary tumour cell line KM12C compared to the highly experimental metastatic cell lines KM12SM and KM12L4a (*p* > 0.05, Figure4D). Interestingly, the 35 kDa isoform was higher expressed in the SW480 cell line than in the SW620 cells.

### Relationship of AEG-1 mRNA and protein expression with clinicopathological variables

**Figure 5:**
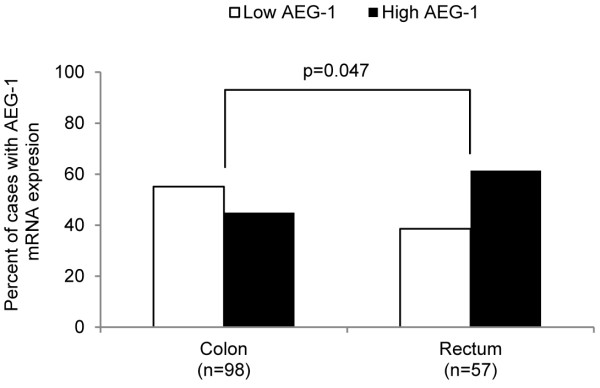
**AEG-1 mRNA expression in the primary tumour compared to the tumour location.** AEG-1 mRNA expression is higher in tumours located in the rectum compared to tumours in the colon (*p* = 0.047).

**Figure 6:**
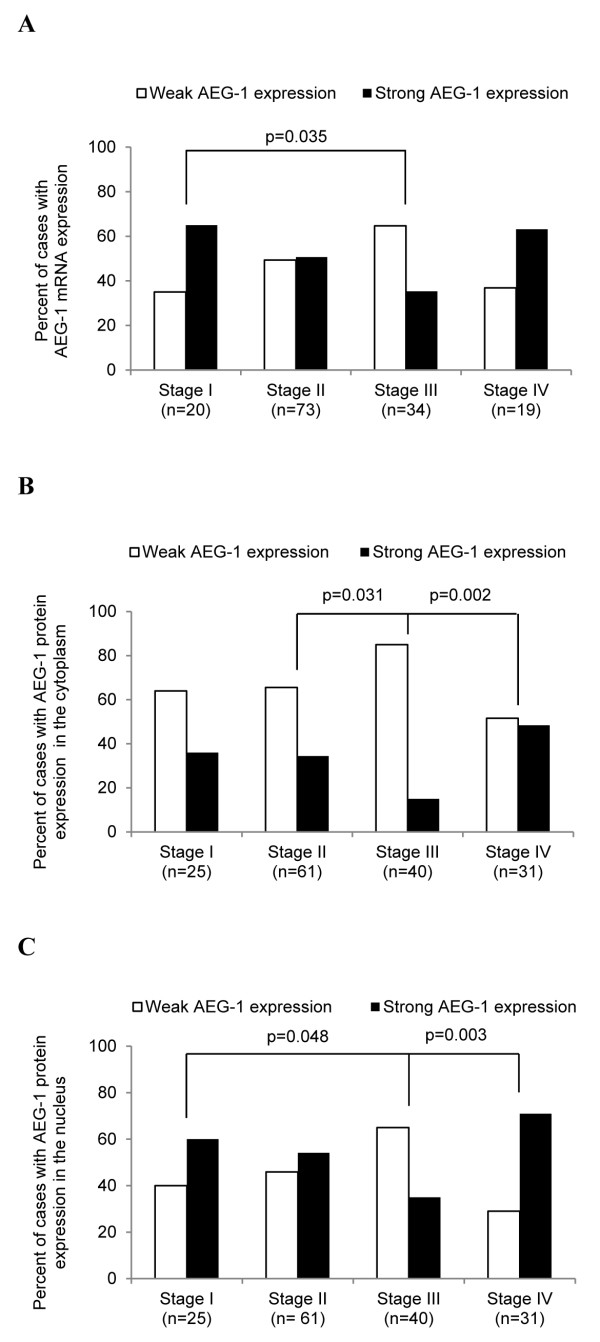
**AEG-1 mRNA and protein expression in the primary tumour compared to tumour stage. (A)** AEG-1 mRNA expression was significantly higher in stage I compared to stage III. **(B)** AEG-1 protein expression in the cytoplasm was similar in stage I and stage II whereas both stages showed a higher staining than stage III (stage I vs. stage II *p* = 0.88; stage I vs. stage III *p* = 0.051; stage II vs. stage III *p* = 0.03), whereas stage III showed a lower staining compared to stage IV (*p* = 0.002). (**C**) AEG-1 protein staining in the nucleus decreased from stage I to stage III (stage I vs. stage II *p* = 0.61; stage I vs. stage III *p* = 0.048; stage II vs. stage III *p* = 0.059), whereas stage III showed a lower staining compared to stage IV (*p* = 0.0026).

We first analysed the relationship of the AEG-1 mRNA expression in primary tumours with clinicopathological variables by dividing the AEG-1 mRNA expression values into two groups (mean value as a cut-off point). AEG-1 expression was significantly higher in the rectal cancer samples compared to colon cancer samples (*p* = 0.047, Figure5). As shown in Figure6A, Chi-square analyses revealed significantly higher AEG-1 expression in stage I tumours compared to stage III tumours (*p* = 0.035). No significant relationship between the AEG-1 mRNA expression, and the gender, age, differentiation or patient survival could be found (*p* > 0.05, Table4).

**Table 4:** AEG-1 mRNA and protein expression in the cytoplasm and nucleus correlated to clinicopathological variables

**Characteristics**	**AEG-1 mRNA expression**			**AEG-1 protein expression in the cytoplasm**			**AEG-1 protein expression in the nucleus**		
	**low (%)**	**high (%)**	***P***	**low (%)**	**high (%)**	***P***	**low (%)**	**high (%)**	***P***
Gender									
Male	54 (52)	49 (48)	0.20	63 (72)	25 (28)	0.24	45 (51)	43 (49)	0.16
Female	22 (42)	31 (58)		44 (63)	26 (37)		28 (40)	42 (60)	
Age (years)									
<70	22 (46)	26 (54)	0.63	40 (68)	19 (32)	0.99	27 (46)	32 (54)	0.93
≥70	52 (50)	52 (50)		67 (68)	32 (32)		46 (46)	53 (54)	
Location									
Colon	54 (55)	44 (45)	0.047	52 (68)	25 (32)	0.96	35 (45)	42 (55)	0.85
Rectum	22 (39)	35 (61)		55 (68)	26 (32)		38 (47)	43 (53)	
Stage									
I	7 (35)	13 (65)	0.28	16 (64)	9 (36)	0.025	10 (40)	15 (60)	0.021
II	36 (49)	37 (51)		40 (66)	21 (34)		28 (46)	33 (54)	
III	22 (65)	12 (35)		34 (85)	6 (15)		26 (65)	14 (35)	
IV	7 (39)	11 (61)		16 (52)	15 (48)		9 (29)	22 (71)	
Differentiation									
Well/moderately	53 (48)	57 (52)	0.64	68 (64)	39 (36)	0.12	46 (43)	61 (57)	0.29
Poorly	21 (53)	19 (48)		38 (76)	12 (24)		26 (52)	24 (48)	

Varied number in the different variables is due to missing data.

In addition, the relationship between AEG-1 protein expression in the primary tumours and the clinicopathological variables was analysed. As shown in Figure6B and 6C, the pattern of AEG-1 protein expression both in the cytoplasm and in the nucleus in different tumour stages were similar to the patterns of the mRNA expression.

Further analysis revealed that AEG-1 protein expression decreased in stage III compared to stage I and stage II, in the cytoplasm (*p* = 0.051, *p* = 0.031, respectively, Figure6B) as well as in the nucleus (*p* = 0.048, *p* = 0.059, respectively, Figure6C). However, the protein expression of AEG-1 was increased in stage IV compared to stage III tumours, both in the cytoplasm as well as in the nucleus (*p* = 0.002 and *p* = 0.003, respectively, Figure6B and 6C). The AEG-1 protein expression was significantly elevated in lymph node metastasis both in the cytoplasm and in the nucleus compared to stage III tumours (*p* = 0.004 and *p* = 0.002, respectively).

There was no relationship between the AEG-1 protein expression and other clinicopathological variables including age, gender, location, differentiation or patient survival (*p* > 0.05, Table4).

### Relationship between AEG-1 protein expression and biological factors

As shown in Table2, cytoplasmic AEG-1 protein expression in the primary tumour was positively correlated to the phosphorylation of NF-κB/p65 at Ser 536 (*p* = 0.002), p73 (*p* = 0.033), and Rad50 (*p* = 0.024) as well as to the apoptotic rate (*p* = 0.006). Nuclear AEG-1 expression was also positively correlated to phosphorylation of NF-κB/p65 at Ser 536 (*p* = 0.034), p73 (*p* = 0.037) and Rad50 (*p* = 0.024) but lost statistical significance with apoptotic rate (*p* = 0.277, Table3).

## Discussion

In the present study, we found a significant increase in AEG-1 mRNA and protein expression in normal mucosa compared to primary tumour. By qPCR we found a 1.5 fold higher AEG-1 mRNA expression in the primary tumour compared to the normal tissue. In concordance, a higher protein expression of AEG-1 was found in the primary tumour compared to the normal mucosa. The immunohistochemical analyses of lymph node metastases revealed a higher expression of AEG-1 compared to the primary tumour even though the difference did not reach statistical significance. Further analyses of liver metastases showed significantly higher AEG-1 expression in the both cytoplasm and nucleus compared to the primary tumour as well as to the lymph node metastases. Moreover we found significantly higher AEG-1 mRNA expression in the metastatic cell line SW620 compared to the parental tumour cell line SW420, and the results were confirmed by Western blot. These results indicate that AEG-1 may promote metastasis and especially distant metastasis.

In the present study, we have seen that the AEG-1 expression in stage III was lower among the different stages. The reason for this is unclear. We speculate that the subclones which were AEG-1 positive within stage III tumour had a high potential to lymph node metastases, leaving a higher amount of AEG-1 negative cells in the primary origin. Supporting this speculation the tumours with higher AEG-1 expression were seen in lymph node metastases compared to primary stage III tumours. This evidence might just occur at the earlier course of metastases, *i.e.*, primary tumour to lymph node metastases, and then to stage IV tumours, AEG-1 may fully unregulated and be involved in distant metastases, such as liver metastases.

By using immunohistochemistry, we found that AEG-1 is expression in the cytoplasm and nucleus of epithelial cells in the normal mucosa as well as in the tumour cells from the primary tumour, lymph node metastasis and liver metastasis. By Western blot we detected the AEG-1 protein at 65, 75 and 35 kDa, respectively, in the colon cancer cell lines. The full length protein consists of a molecular weight of about 65 kDa whereas the 75 kDa protein corresponds to the mono-ubiquitinated form of AEG-1, in addition the 35 kDa protein corresponds to the alternatively spliced variant of AEG-1 localised mainly in the nucleus [15]. Earlier studies by Song *et al.* (2010) detected AEG-1 as the full length, 65 kDa protein, together with the 75 kDa mono-ubiqitinated isoforms localised in the nucleus in only a few primary tumours and in liver metastasis in Chinese CRC patients [10]. Furthermore they found increased AEG-1 expression from stage I towards stage IV tumours, and a significant correlation between high AEG-1 expression and poor patient survival [10]. The differences in the expression pattern and the localisation between the studies might be due to different target epitopes of the antibodies, leading to the staining of different AEG-1 isoforms [15].

Since our present study shows that the expression of the 65 kDa, and the 75 kDa subunits was higher at the same time as the 32 kDa subunit was lowest in the metastatic SW620 cell line compared to the primary tumour cells SW480, we suggest these isoforms to be differently regulated during CRC development.

In addition, an explanation for the discrepancies between our study and previous studies could be due to the different ethnical background of the patients analysed [28]. Cancer development and genomic alterations are known to be different between Caucasian and other ethnical groups [29,30]. Variations in AEG-1 localisation due to different ethnicities have also been shown in breast cancer. A Chinese study analysed 225 breast cancer patients demonstrated a more nuclear AEG-1 staining in metastatic tumours [14], whereas in a separate study analysing North American patients, AEG-1 was predominantly localised to the cytoplasm analysed in 170 tissue sections of human breast cancer [31]. Therefore it would be of interest to investigate the function and expression of the different AEG-1 isoforms in patients with different ethnical backgrounds.

Moreover, we found significantly higher AEG-1 mRNA expression in the primary tumour of the rectum compared the colon, indicating that AEG-1 is differently expressed at these two sites. There has been an extensive debate about the similarity and diversity of the disease. Even though rectal cancer is similar to colon cancer, it exhibits its own specific features genetically as well as physiologically. The colon, for instance, originates from the midgut and hindgut and the rectum from the cloaca. The major physical function also differs, whereas the colon absorb nutrients and water, the function of the rectal is to store faeces [32]. According to molecular pathways rectal cancer shows more nuclear β-catenin and a higher COX-2 protein level than colon cancer, while rectal cancer is less *K-ras*-dependent [33,34]. Even the most intensively studied tumour suppressor p53 shows higher expression in rectal compared to colon tumours [33]. The differences in the AEG-1 mRNA expression between colon cancer and rectal cancer support the idea of different entities.

AEG-1 was originally identified as a HIV-1 - inducible gene in human fetal astrocytes [35]. Lee et al. (2006) published the first activation pathway of AEG-1 in which AEG-1 is activated by the oncogene Ha-Ras possibly through the activation of the PI3K/Akt pathway that causes c-Myc binding to the E-box element of AEG-1 promoter [18]. A possible interaction partner of AEG-1 was found by Emdad et al. (2006), who reported that AEG-1 promotes anchorage-independent growth and invasion through IκBα degradation, NF-κB binding and nuclear translocation [19]. Furthermore, several studies demonstrated a functional role of AEG-1 in several aspects of cancer development including angiogenesis, invasion, metastasis, apoptosis and chemo-resistance [7,11,36,37]. In the present study, we found several correlations of the AEG-1 protein expression in the primary tumour to other biological factors. Both cytoplasmic and nuclear AEG-1 expression were significantly correlated to the phosphorylation of NF-κB/p65 at Ser 536. It has been reported that the phosphorylation of NF-κB/p65 at Ser 536 is induced by the IkB kinases α and β, which are activated by the tumour necrosis factor-α, leading to p65 translocation to the nucleus [38]. Several lines of evidence have suggested that AEG-1 activates NF-κB via degradation of IκBα and by direct binding on the p65 subunit [19,39]. Our data suggest an AEG-1-NF-κB interaction in CRC, but further details about the signalling pathways involved remain to be investigated in future studies.

**Figure 7:**
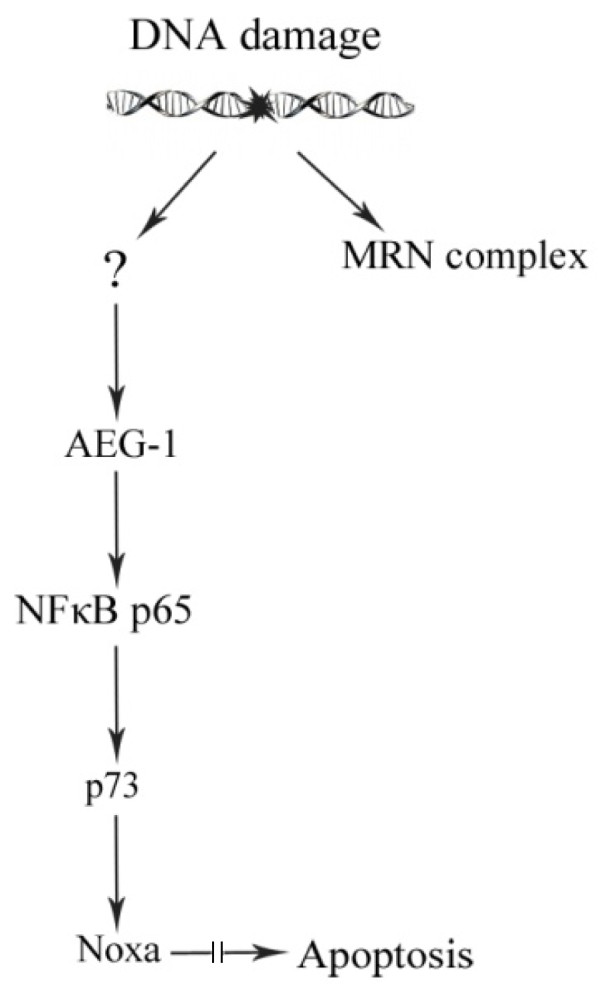
**Possible involvement of AEG-1 in a p53 independent apoptotic pathway.** After DNA-damage the MRN complex is recruited to the DNA break. AEG-1 is activated after DNA damage by an unknown mechanism, leading to NF-κB activation. The active p65 subunit of NF-κB is required for p73 dependent Noxa gene expression, leading to apoptosis.

Moreover, we showed, for the first time, a positive correlation of the cytoplasmic and the nuclear AEG-1 expression to p73 and Rad50 as well as a positive correlation between cytoplasmic AEG-1 and the apoptotic rate. Martin et al. (2009) discovered a p53 independent apoptotic pathway after DNA damage in mouse fibroblasts. In their study, p73 induces Noxa gene expression after genotoxin treatment only in the presence of the NF-κB subunit p65 independently of p53 [40]. In light of our findings, we speculate that AEG-1 may participate in the signal cascade of this p53 independent apoptotic pathway which would explain the correlation to Rad50, p73, NF-κB/p65 at Ser 536 and apoptosis seen in our study (Figure7). Supporting this is the finding by Su et al. (2010) about a correlation between high AEG-1 expression and mutated p53 determined by using immunohistochemistry since positive p53 immunohistochemical staining is representative of mutated p53 due to its long half-life [41,42]. Taken their and our results together it seems that, after DNA damage, AEG-1 may be activated by an unknown mechanism, leading to p65 activation, and this step might be crucial for the p73 (instead of wild-type p53) dependent Noxa expression, leading to apoptosis. In addition, it is known that after DNA damage, amongst others, the MRN complex is recruited to the DNA break by the factor ataxia telangiectasia mutated (ATM) essential for DNA double strand break repair. ATM together with the IKKγ/NF-κB essential modulator (NEMO) degrades IκBα and activates NF-κB in response to DNA-damage [43].

## Conclusion

AEG-1 expression at the mRNA and/or protein level was up-regulated during the CRC development and aggressiveness from normal mucosa to primary tumour, to lymph node metastasis and to liver metastasis. The AEG-1 mRNA expression was higher in tumours located in the rectum than in those located in the colon. Furthermore our study suggests that AEG-1 interacted with NF-κB in CRC and may thereby be involved in a p53 independent apoptotic pathway after DNA damage.

## Abbreviation

AEG-1: Astrocyte elevated gene 1; CRC: Colorectal cancer; MTDH: Metadherin; HIV-1: Human immunodeficiency virus-1; NF-κB: Nuclear factor kappa B; IκBα: NF-κB inhibitor α; EMT: Epithelial mesenchymal transition; ATM: Ataxia telangiectasia mutated.

## Competing interests

The authors declare that they have no competing interests.

## Authors’ contributions

SG carried out the qPCR, immunohistochemistry, and Western blot studies, performed the statistical analyses, participated in the study design and drafted the manuscript. YMS participated in the qPCR and immunohistochemistry studies, the statistical analyses and the study design. CJW participated in the immunohistochemistry study. HZ participated in the immunohistochemistry study. JS participated in the qPCR studie. GA contributed the patient material with clinical data. XFS conceived of the study and participated in its design and coordination and helped to draft the manuscript. All authors read and approved the final manuscript.
